# Commentary: Excessive Iodine Promotes Pyroptosis of Thyroid Follicular Epithelial Cells in Hashimoto's Thyroiditis Through the ROS-NF-κB-NLRP3 Pathway

**DOI:** 10.3389/fendo.2020.00581

**Published:** 2020-08-28

**Authors:** Yuji Nagayama

**Affiliations:** Department of Molecular Medicine, Atomic Bomb Disease Institute, Nagasaki University, Nagasaki, Japan

**Keywords:** thyroid cell, iodine uptake, Nthy-ori 3-1, PCCL3, sodium-iodine symporter

It is well-known that iodine is an indispensable element for thyroid hormone synthesis, and both deficiency and excess in iodine cause thyroid dysfunction. The article mentioned above used a human normal thyroid cell line, Nthy-ori 3-1, to study the effect of excessive iodine on thyroid cell death (1), where cell death was induced by incubating the cells with extremely high concentrations of iodine (≥10^−2^ M). The same combination of Nthy-ori 3-1 cells and very high concentrations of iodine (up to 5 × 10^−2^ M) has also been previously used for studies on iodine-induced thyroid cell death, ER stress induction, etc. (2, 3).

I would like to raise two issues about this article. First is about the cell's capability of iodine uptake. The previous articles report that Nthy-ori 3-1 cells do not incorporate iodine (4, 5). Nthy-ori 3-1 cells were derived from HTori-3 cells which are immortalized human thyroid cells by transfection of simian virus 40 T-antigen (https://web.expasy.org/cellosaurus/CVCL_2659), and can grow without TSH. Thus, it is easy to assume that these cells are rather de-differentiated. In contrast, rat functional thyroid cell lines, FRTL5 and PCCL3, both of which are spontaneously immortalized cell lines and their growth is totally TSH-dependent, a characteristic of differentiated thyroid cells, take up iodine. We recently confirmed that PCCL3 cells incorporated ^131^I in a dose-dependent manner, reaching the peak at 30 min in the presence of TSH (2 mU/mL), which was completely abolished by 5 μM NaClO_4_ (perchlorate), a competitive inhibitor of sodium-iodine symporter (NIS), confirming NIS-mediated iodine uptake (6). Thus, I am afraid that the effect of iodine on Nthy-ori 3-1 cell death observed in (1) does not appear to be exerted by iodine incorporated into the cells.

Second is the concentration of iodine used. The physiological concentration of iodine in human sera is typically 2–6 × 10^−8^ M (5–15 μg/L) (7), and pretreatment with 10^−6^ M or more iodine suppresses subsequent iodine uptake and expression of NIS (i.e., Wolff-Chaikoff effect) in FRTL5 cells (8) and porcine thyroid cells in suspension culture (9). 10^−3^ M iodine is generally used as an excessive dose in FRTL5 and PCCL3 cells, which suppresses the expression of other thyroid differentiation genes such as thyrotropin receptor, thyroglobulin, and thyroid peroxidase (10–12), and induces pendrin expression/iodine efflux (13). Thus, the concentration of iodine used in (1) is 10^6^ times higher than the physiological serum concentration, 10^4^ times higher than that required for the Wolff-Chaikoff effect, and ≥10 times higher than the excess dose used in FRTL5 and PCCL3 cells.

Despite of a clear difference in the ability of iodine uptake between FRTL5 and Nthy-ori 3-1 as mentioned above, cell death can only be induced by very high doses of iodine (≥10^−2^ M) in both cells (1, 14). The ability of iodine uptake and cytotoxicity of iodine are summarized in the Table 1. In addition, human thyroid cells cultured in the presence of 1 mU/mL TSH are resistant to iodine-induced cell death with up to 3 × 10^−2^ M iodine for 24 h (15). Therefore, thyroid cells used in all the above reports are relatively resistant to a high dose of iodine, irrespective of their capability of iodine uptake. It is at present unknown as to how extremely high concentrations of iodine in the culture medium exert its cell killing effect in Nthy-ori 3-1 cells. Of interest, a recent paper has described the impairment of reproductive function in male rats by excess iodine, which is thought to be attributed to the aberrant expression of NIS in the testis (16).

**Table 1:** Summary of iodine uptake and cytotoxicity of iodine in FRTL5/PCCL3 cells and Nthy-ori 3-1 cells.

	**Iodine uptake**	**References**	**Cytotoxicity of iodine for 24 h**	**References**
FRTL5	Yes	(8)	Yes, ≥ 3 × 10^−2^ M	(15)
Nthy-ori 3-1	No	(4, 5)	Yes, ≥ 10^−2^ M	(1)

Altogether, although it is understandable that one prefers to use the thyroid cells of human origin rather than of rodent origin, I would like to express my concern that Nthy-ori 3-1 cells may not be suitable for studies on the *in vitro* effect of iodine on thyroid cell function and/or survival.

## Author Contributions

YN has written the manuscript.

## Conflict of Interest

The author declares that the research was conducted in the absence of any commercial or financial relationships that could be construed as a potential conflict of interest.

## References

[1] Liu J Mao C Dong L Kang P Ding C Zheng T. Excessive iodine promotes pyroptosis of thyroid follicular epithelial cells in hashimoto's thyroiditis through the ROS-NF-κB-NLRP3 pathway. Front Endocrinol. (2019) 10:778. 10.3389/fendo.2019.00778 31824415 PMC6880659

[2] Liu H Zeng Q Cui Y Yu L Zhao L Hou C. The effects and underlying mechanism of excessive iodide on excessive fluoride-induced thyroid cytotoxicity. Environ Toxicol Pharmacol. (2014) 38:332–40. 10.1016/j.etap.2014.06.008 25104093

[3] Liu H Zeng Q Cui Y Zhao L Zhang L Fu G. The role of the IRE1 pathway in excessive iodide- and/or fluoride-induced apoptosis in Nthy-ori 3-1 cells *in vitro*. Toxicol Lett. (2014) 224:341–8. 10.1016/j.toxlet.2013.11.001 24231001

[4] Tuncel M Aydin D Yaman E Tazebay UH Güç D Dogan AL. The comparative effects of gene modulators on thyroid-specific genes and radioiodine uptake. Cancer Biother Radiopharm. (2007) 22:443–9. 10.1089/cbr.2006.319.A 17679169

[5] Lemoine NR Mayall ES Jones T Sheer D McDermid S Kendall-Taylor P. Characterisation of human thyroid epithelial cells immortalised *in vitro* by simian virus 40 DNA transfection. Br J Cancer. (1989) 60:897–903. 10.1038/bjc.1989.387 2557880 PMC2247263

[6] Kurashige T Shimamura M Nagayama Y. N-Acetyl-L-cysteine protects thyroid cells against DNA damage induced by external and internal irradiation. Radiat Environ Biophys. (2017) 56:405-412. 10.1007/s00411-017-0711-8 28871381

[7] Iodine and Inorganic Iodides: Human Health Aspects (CICADS 72) (2009). Available online at: http://www.inchem.org/pages/cicads.html

[8] Grollman EF Smolar A Ommaya A Tombaccini D Santisteban P. Iodine suppression of iodide uptake in FRTL-5 thyroid cells. Endocrinology. (1986) 118:2477–82. 10.1210/endo-118-6-2477 3009160

[9] Takasu N Handa Y Kawaoi A Shimizu Y Yamada T. Effects of iodide on thyroid follicle structure and electrophysiological potentials of cultured thyroid cells. Endocrinology. (1985) 117:71–6. 10.1210/endo-117-1-71 4006867

[10] Leoni SG Galante PA Ricarte-Filho JCM Kimura ET. Differential gene expression analysis of iodide-treated rat thyroid follicular cell line PCCl3. Genomics. (2008) 91:356–66. 10.1016/j.ygeno.2007.12.009 18272324

[11] Serrano-Nascimento C Calil-Silveira J Goulart-Silva F Nunes MT. New insights about the posttranscriptional mechanisms triggered by iodide excess on sodium/iodide symporter (NIS) expression in PCCl3 cells. Mol Cell Endocrinol. (2012) 349:154–61. 10.1016/j.mce.2011.09.036 22001309

[12] Eng PH Cardona GR Previti MC Chin WW Braverman LE. Regulation of the sodium iodide symporter by iodide in FRTL-5 cells. Eur J Endocrinol. (2001) 144:139–44. 10.1530/eje.0.1440139 11182750

[13] Calil-Silveira J Serrano-Nascimento C Kopp PA Nunes MT. Iodide excess regulates its own efflux: a possible involvement of pendrin. Am J Physiol Cell Physiol. (2016) 310:C576–82. 10.1152/ajpcell.00210.2015 26791486 PMC4971809

[14] Golstein J Dumont JE. Cytotoxic effects of iodide on thyroid cells: difference between rat thyroid FRTL-5 cell and primary dog thyrocyte responsiveness. J Endocrinol Invest. (1996) 19:119–26. 10.1007/bf03349847 8778164

[15] Vitale M Di Matola T D'Ascoli F Salzano S Bogazzi F Fenzi G. Iodide excess induces apoptosis in thyroid cells through a p53-independent mechanism involving oxidative stress. Endocrinology. (2000) 141:598–605. 10.1210/endo.141.2.7291 10650940

[16] Chakraborty A Mandal J Mondal C Sinha S Chandra AK. Effect of excess iodine on oxidative stress markers, steroidogenic-enzyme activities, testicular morphology, and functions in adult male rats. Biol Trace Elem Res. (2016) 172:380–94. 10.1007/s12011-015-0581-3 26701334

